# Predictors of Mental Health Review Tribunal (MHRT) outcome in a forensic inpatient population: a prospective cohort study

**DOI:** 10.1186/s12888-016-1188-8

**Published:** 2017-01-17

**Authors:** Amelia Jewell, Kimberlie Dean, Tom Fahy, Alexis E. Cullen

**Affiliations:** 1 Institute of Psychiatry, Psychology & Neuroscience, King’s College London, De Crespigny Park, London, SE5 8AF UK; 2 South London and Maudsley NHS Foundation Trust, London, UK; 3 School of Psychiatry, Faculty of Medicine, University of New South Wales, Sydney, Australia; 4 Justice Health & Forensic Mental Health Network, Matraville, NSW Australia

**Keywords:** Mental Health Review Tribunal, First Tier Tribunal, Forensic inpatient, Medium secure unit, Mentally disordered offender

## Abstract

**Background:**

Previous studies have investigated factors associated with outcome at Mental Health Review Tribunals (MHRTs) in forensic psychiatric patients; however, dynamic variables such as treatment compliance and substance misuse have scarcely been examined, particularly in UK samples. We aimed to determine whether dynamic factors related to behaviour, cooperation with treatment, and activities on the ward were prospectively associated with outcome at MHRT.

**Methods:**

At baseline, demographic, clinical, behavioural, and treatment-related factors were ascertained via electronic medical records and census forms completed by the patient’s clinical team. Data on MHRTs (i.e., number attended, responsible clinician’s recommendation, and outcome) were collected at a 2-year follow-up. Logistic regression analyses were performed to determine factors associated with outcome among those who attended a MHRT within the follow-up period. Of the 135 forensic inpatients examined at baseline, 79 patients (59%) attended a MHRT during the 2-year follow-up period and therefore comprised the study sample. Of these 79 patients included in the current study, 28 (35%) were subsequently discharged.

**Results:**

In univariable analyses, unescorted community leave, responsible clinician’s recommendation of discharge, and restricted Mental Health Act section were associated with a greater likelihood of discharge at MHRT; whilst inpatient aggression, a recent episode of acute illness, higher total score on the Historical Clinical Risk – 20 (HCR-20), higher HCR-20 clinical and risk scores, and agitated behaviour were negatively associated with discharge (*p* < 0.05). In multivariable analyses, HCR-20 clinical scale scores and physical violence independently predicted outcome at tribunal after controlling for other dynamic variables.

**Conclusion:**

By identifying dynamic factors associated with discharge at tribunal, the results have important implications for forensic psychiatric patients and their clinical teams. Our findings suggest that by reducing levels of agitated behaviour, verbal aggression, and physical violence on the ward, achieving unescorted community leave, and targeting specific items on the HCR-20 risk assessment tool, patients may be able to improve their changes of discharge at a MHRT.

## Background

In the United Kingdom (UK), Mental Health Review Tribunals (MHRTs: renamed First-Tier Tribunals in 2008) are independent panels that review compulsory treatment orders for individuals with mental illness [1]. Known in the UK, Ireland, Australia, and New Zealand as MHRTs or Mental Health Tribunals, elsewhere in the world, similar review panels are referred to as Mental Health Review Boards (Canada) or Psychiatric Review Boards (Japan) [2]. In other European countries, involuntary admissions are typically reviewed by a judge rather than a specialist tribunal [3], whilst the United States of America (USA) follow a purely judicial model with Civil Commitment Proceedings held before a court [4].

Whilst the function of MHRTs may vary from country to country, all tribunals act as a safeguard to ensure that the continued detention of a patient under psychiatric care is not unlawful. MHRTs in the UK function under the current Mental Health Act 1983 (MHA) legislation. The tribunal panel — typically consisting of a medical member, a tribunal judge, and a lay member [5–7] — have the power to uphold the detention of a patient in psychiatric hospital or direct their discharge, either conditionally, in the case of restricted patients (i.e., those patients held on restriction orders directed by the Crown Court due to the risk that they pose to the public), or absolutely [5]. Applications to the MHRT are made by the patient or a close relative and the point at which an appeal can be made is dependent on the jurisdiction and admission status under which the patient is detained. In the UK, any patient who has not attended a tribunal within the past 3 years is automatically referred by the Secretary of State [5].

Since their introduction, the number of appeals to the MHRT in England and Wales has risen steadily [8], from 904 in 1980 [9] to 31,469 in 2014 [10], likely reflecting the parallel increase in the number detained. This is in contrast to New Zealand and Ireland where the number of MHRTs has remained relatively constant since their introduction [11, 12]. However, the percentage of patients who are successful in obtaining discharge at MHRT hearings is relatively low across the UK and abroad [11–14]. In the UK, the Care Quality Commission (CQC) reported that only 9% of all hearings in 2013/14 resulted in discharge [15], with the likelihood of discharge being greater among restricted patients than unrestricted patients (22% vs. 10%, respectively [15]). Indeed, MHRTs are the principal avenue for discharge for restricted patients in England and Wales, whereas unrestricted patients are more likely to be simply discharged by their treating team. In 2009, 68% of all discharges of restricted patients to the community were via conditional discharge by tribunal [16]. Despite this, relatively little is known about the factors which influence outcome decisions at tribunal.

Previous studies examining factors associated with outcome at MHRT hearings have tended to examine heterogeneous samples comprising both forensic patients, that is, those patients who have been detained under Part III of the MHA (i.e., patients concerned in criminal proceedings or under sentence), and civil patients. These studies demonstrate that with regards to demographic factors, reassuringly, neither ethnicity nor age are associated with outcome at tribunal [13, 14, 17–21]. The role of patient sex is unclear; trend-level associations reported in samples of both civil and forensic patients in both the UK and New Zealand indicate that females are more likely to be discharged than males [6, 13, 22]; however, the only previous study to have found a significant association between sex and outcome reported that males are more likely to receive discharge at tribunal than females [17]. Findings with regard to diagnosis are also mixed, Dibben and colleagues reported that when compared to patients with schizophrenia, those with mania were five times more likely to be discharged by the tribunal [23], yet other studies have observed that neither mental disorder [17, 20, 21] nor personality disorder [18] are related to outcome at MHRT. Perhaps unsurprisingly, a study conducted in a sample of maximum security inpatients in Canada reported that patients with higher psychopathy scores were significantly less likely to be discharged than those with lower scores [7]. In contrast, in a forensic (high-security) hospital sample in the UK it was reported that individuals with psychopathic disorder were more likely to be discharged in comparison to patients diagnosed with mental illness [13].

Whilst the aforementioned studies have identified a number of static factors that may influence outcome at tribunal, a qualitative study of 50 tribunals in the UK (including both forensic and civil patients) reported that when making a decision on outcome, tribunal panels ask questions about dynamic variables (i.e., those that are potentially amenable to change) such as behaviour, cooperation with treatment, substance misuse, and activities on the ward [24]. Yet only three previous studies have attempted to explore the relationship between dynamic factors and outcome at MHRT. These studies, based in Canada and Ireland, reported that institutional management problems, treatment compliance, active psychotic symptoms, treatment success [7, 25], and scores on the Historical Clinical Risk-20 (HCR-20), a widely-used violence risk assessment tool [21], were all associated with outcome at tribunal. These findings are yet to be replicated within a UK sample and there are a number of potentially important dynamic factors, such as violent or sexually inappropriate behaviour, and leave status, which have not yet been explored in the literature.

Identifying dynamic factors associated with success at MHRTs could provide patients and clinical teams with ways to improve a patient’s chances of discharge, potentially reducing the number (and subsequent cost) of unsuccessful appeals. Thus, previous studies examining static variables, which, by definition, are not amenable to change, are limited in the extent to which they can provide clinically useful findings. Moreover, the fact that previous studies examining dynamic factors associated with outcome at MHRT have been retrospective in nature introduces the possibility of reverse causality (i.e., where knowledge of the outcome may bias the way in which exposure data is collected). Further to this, the majority of previous studies have looked at civil and forensic psychiatric patients together, making it difficult to draw conclusions regarding forensic patients specifically, the majority of which are held under restriction orders (in 2014/15 the Health and Social Care Information Centre (HSCIC) reported that 70% of patients detained under Part III of the MHA were subject to a restriction order [26]). This is particularly important because tribunals are the principal method for discharge into the community for restricted patients in England and Wales [16].

The current study utilised data from a longitudinal study of a sample (*N* = 135) of forensic psychiatric inpatients in the UK [27]. Given the paucity of research examining dynamic factors, particularly in forensic populations, the primary aim of the current study was to examine the extent to which dynamic factors related to recent inpatient behaviour, cooperation with treatment, and substance misuse are associated with outcome at tribunal in this population.

## Methods

### Sample and setting

The current study utilises data from a longitudinal study [27] of forensic psychiatric inpatients within the South London and Maudsley (SLaM) National Health Service (NHS) Foundation Trust. SLaM is one of Europe’s largest providers of secondary mental health care and provides care predominately for the London boroughs of Lambeth, Southwark, Croydon, and Lewisham [28]. In November 2011, an initial census was conducted of all SLaM patients receiving treatment in forensic inpatient services during a 2-week period [27], including those who were admitted or discharged during this time. At baseline, SLaM’s forensic inpatient services consisted of two medium-secure units (comprising eight inpatient wards) and one low-secure unit consisting of a single ward. In total, the sample consisted of 135 individuals, both male and female, aged 18 to 67.

### Data collection

Census data were collected using the Biomedical Research Centre (BRC) Clinical Records Interactive Search (CRIS) tool, an anonymised database of electronic medical records. The CRIS database, described in detail previously [28–30], provides access to anonymised electronic medical records for over 250,000 mental health service users within the SLaM NHS Foundation Trust [30]. Records are passed through a de-identification process that removes the name, date of birth (retaining month and year), and address (retaining first half of the postcode) from both the structured and unstructured fields within the records [31]. The CRIS database allows users to extract data located within both the structured and unstructured fields of the medical records and has the advantage that free text searching can be used to identify relevant documents and entries relating to specific keywords or phrases.

The aim of the census was to obtain detailed demographic, clinical, behavioural, and treatment-related data for a representative sample of forensic inpatients, and to follow this cohort longitudinally. A census form was designed to capture clinical and forensic variables, such as inpatient violence and sexually inappropriate behaviour, which were not systematically recorded in medical records. Census forms were completed by the multidisciplinary treating team for each inpatient receiving treatment and uploaded to the electronic medical records system; data were subsequently extracted by the research team using the CRIS database, thus retaining anonymity.

Following a detailed literature review, a number of factors from the original census were chosen to examine within the current study, these were separated into four categories; (i) demographic, (ii) clinical, (iii) behavioural, and (iv) treatment-related factors. Demographic factors (sex, date of birth, and ethnicity) were extracted using CRIS from structured fields within the medical records, and MHA section and current leave status were ascertained via the census form. With regards to clinical factors, primary diagnosis was determined from the most recent psychiatric report available via CRIS. Additionally, clinicians were asked to rate the presence of personality disorder (primary or co-morbid diagnosis) and psychopathy as ‘not present’, ‘present but not formally assessed’, or ‘present and formally assessed using a validated tool’ on the census form. Validated tools to assess personality disorder included the Minnesota Multiphasic Personality Inventory (MMPI) [32] and the Structured Clinical Interview for DSM-IV Axis II Personality Disorders (SCID-II) [33], and for psychopathy, the Psychopathic Checklist Revised (PCL-R) [34] and Psychopathic Checklist Screening Version (PCL-SV) [35]. The census form was also used to establish whether the patient had experienced an episode of acute illness in the past 12 months, this included depression, psychosis, and mania.

Behavioural factors were largely identified using the census form. Specifically, the census form included questions to determine the primary index offence and whether the patient had displayed inappropriate sexual behaviour, aggressive behaviour, agitated behaviour, or used substances in an inpatient setting in the 12 months prior to the census. The clinical team were additionally asked to indicate whether the patient had ever absconded or escaped whilst detained in an inpatient setting. Free text searching within the CRIS database was also used to identify the most recent (proximal to the census) HCR-20 report for each patient [36]. Finally, clinician reported treatment-related factors were ascertained via the census form, including regular medication (particular attention was paid to Clozapine and depot antipsychotic use), and information pertaining to a history of medication compliance issues and engagement in psychological therapy. Psychological therapies attended by the patients within the cohort included the Violence Reduction Programme (VRP), HCR-20 Risk and Recovery Group, Managing Mental Health, and Aggression Replacement Training. As patients often participated in a number of different interventions, the effect of these interventions on outcome were not individually examined.

### Outcome measure

Outcome data were extracted via CRIS approximately 24 months after census completion. Details of all MHRTs, including (i) number attended, (ii) responsible clinicians’ recommended outcome, (iii) date, and (iv) outcome, were ascertained for all patients in the cohort. The free text search tool within CRIS was used to search the ‘ward progress notes’, ‘correspondence’, and ‘events’ sections within the electronic medical records (i.e., unstructured fields containing entries made by the clinical teams at multiple points throughout the day) in order to identify relevant documents. A pilot search was conducted to determine the search terms and output fields required to bring back relevant records and subsequently ‘MHRT’, ‘tribunal*’, ‘report’, ‘review*’, ‘MHT’, ‘MHA tribunal’, ‘appeal*’, ‘decision*’, and ‘discharge*’ were used for the final search terms. Retrieved entries were then manually cleaned and for those individuals who had attended a tribunal during the follow-up period, two binary variables were created for each MHRT attended; (i) the responsible clinicians recommendation, (coded as detain vs. discharge) and (ii) outcome of the tribunal (discharged vs. section upheld). Only data on the first MHRT attended by each patient within the follow-up period were included in statistical analyses.

### Statistical analyses

Data were analysed using IBM Statistical Package for Social Scientists (SPSS) version 21 for Windows. Univariable logistic regression analyses were first performed on all demographic, clinical, behavioural, and treatment-related factors to examine the association with MHRT outcome; these analyses were performed on a subsample of the total cohort (i.e., only those who attended a MHRT within the follow-up period). In order to determine which significant dynamic variables uniquely predicted outcome at MHRT, dynamic variables found to be statistically significant at the *p* < 0.01 level in univariable analyses were advanced to the multivariable analysis stage. These variables were first checked for multicollinearity, and any outliers in the sample were additionally identified and removed. Multivariable logistic regression was then performed to identify factors which remained significant predictors of outcome after controlling for all other significant dynamic variables.

## Results

### Sample demographics

Within the total cohort (*N* = 135), 79 (59%) patients attended a MHRT hearing during the follow-up period, of these, 12 (15%) patients attended two tribunals and two (3%) patients attended three tribunals during the follow-up period. The median length of time to the first tribunal was 222 days (range: 6–749). For the patients who attended a tribunal during the follow-up period, the mean age at baseline was 38.1 years (SD 11.9). The majority of patients who attended a tribunal were male (93.7%) and most patients (71.8%) were being held on a restricted MHA section (sections 37/41, 47/49, and 48/49) at baseline. Approximately three-quarters (77.8%) of the patients who attended a tribunal had a primary diagnosis of psychotic disorder (i.e., schizophrenia, schizoaffective, or other psychotic disorder). At baseline, the median length of stay for the current inpatient episode was 505 days (range: 24–2,510). Univariable logistic regression analysis was conducted to compare those patients who attended a MHRT during the follow-up period and those who did not. Analyses revealed no statistically significant difference in demographic factors including age, sex, ethnicity, or MHA section (all *p* values > 0.05). Of the 79 patients who attended a MHRT, 28 (35.4%) were discharged, whilst 51 (64.6%) had their sections upheld.

### Univariable analysis of predictors of outcome at MHRT

Results of univariable logistic regression analyses examining the association between demographic factors and outcome at MHRT are presented in Table 1. Patients held on a restricted section (sections 37/41, 47/49, and 48/49) were significantly more likely to be discharged by the MHRT tribunal relative to those held on an unrestricted section (OR = 16.47; 95% CI = 2.06, 131.59; *p* = 0.008); however, sex, age, and ethnicity were not significant predictors of outcome (*p* > 0.05). In terms of leave status, relative to patients with no leave granted, the odds of being discharged by the tribunal were higher among those who had any form of leave at study commencement. This was only statistically significant for patients who had been granted unescorted community leave at baseline (OR = 12.36; 95% CI = 2.37, 64.64; *p* = 0.003), with 64% of patients who were discharged having unescorted community leave compared to only 22% among those whose section was upheld.

**Table 1:** Logistic Regression analyses examining demographic and clinical predictors of outcome at MHRT

Predictor	Section Upheld (*n* = 51)	Discharged (*n* = 28)	OR	(95% CI)	*P* Value
Demographic factors					
Age (years): mean (SD)	38.8 (11.5)	36.9 (12.6)	0.99	(0.95 to 1.03)	0.48
Female sex: *n* (%)	2 (3.9)	3 (10.7)	2.94	(0.46 to 18.75)	0.25
Ethnicity: *n* (%)					
White British	13 (26.0)	6 (23.1)	(ref)	-----	-----
African or Caribbean	14 (28.0)	11 (42.3)	1.70	(0.49 to 5.93)	0.40
Other	23 (46.0)	9 (34.6)	0.85	(0.25 to 2.92)	0.79
Restricted MHA section: *n* (%)	30 (61.2)	26 (96.3)	16.47	(2.06 to 131.59)	0.008
Leave status: *n* (%)					
No leave	17 (34.0)	2 (8.0)	(ref)	-----	-----
Ground leave	4 (8.0)	2 (8.0)	4.25	(0.45 to 40.01)	0.21
Escorted community leave	18 (36.0)	5 (20.0)	2.36	(0.40 to 13.84)	0.34
Unescorted community leave	11 (22.0)	16 (64.0)	12.36	(2.37 to 64.64)	0.003
Clinical factors					
Diagnosis: *n* (%)					
Schizophrenia, schizoaffective, and other psychotic disorder	41 (80.4)	21 (75.0)	(ref)	-----	-----
PD, bipolar, and other	10 (19.6)	7 (25.0)	1.37	(0.46 to 4.11)	0.59
PD present: *n* (%)					
No	21 (42.9)	16 (59.3)	(ref)	-----	-----
Possible/likely	14 (28.6)	3 (11.1)	0.28	(0.07 to 1.15)	0.08
Yes	14 (28.6)	8 (29.6)	0.75	(0.25 to 2.22)	0.60
Psychopathy present: *n* (%)	12 (24.5)	4 (15.4)	0.56	(0.16 to 1.95)	0.36
Recent acute illness: *n* (%)	21 (42.9)	4 (15.4)	0.24	(0.07 to 0.81)	0.02
RC recommended discharge: *n* (%)	3 (6.3)	18 (66.7)	30.00	(7.28 to 123.66)	<0.001

Note. OR: odds ratio; *n*: subgroup sample size; CI: confidence interval; SD: standard deviation; MHA: Mental Health Act; PD: personality disorder; RC: responsible clinician. Acute illness: includes depression, mania, and psychosis. Missing data: ethnicity (*n* = 3); restricted MHA section (*n* = 3); leave status (*n* = 4); PD present (*n* = 2); psychopathy present (*n* = 2); recent episode of acute illness (*n* = 4); RC recommended discharge (*n* = 4). For all variables, ‘current’ and ‘recent’ is relative to the time of the initial census

Of the clinical factors examined (Table 1), neither primary diagnosis, personality disorder (primary or co-morbid diagnosis) nor psychopathy were significant predictors of outcome (*p* > 0.05). However, patients who had experienced a recent episode of acute illness (depression, mania, or psychosis) at the start of the study were significantly less likely to be discharged by the tribunal compared to those who had not been recently unwell (OR = 0.24; 95% CI = 0.07, 0.81; *p* = 0.02). With regards to the responsible clinicians’ recommendation, the odds of being discharged were 30 times greater when the patients’ responsible clinician recommended discharge in comparison to when the clinician recommended that the MHRT uphold the patients section. This finding was highly statistically significant (OR = 30.00; 95% CI = 7.28, 123.66; *p* < 0.001), although the odds ratio was associated with a wide confidence interval.

Behavioural factors and their association with outcome are presented in Table 2. Analyses indicated that neither index offence, substance use in an inpatient setting, nor inappropriate sexual behaviour (both latter items rated within the 12 months period preceding the initial census) were significant predictors of outcome (*p* > 0.05). Substantially fewer patients who had been discharged had a history of absconsion compared to those who had their section upheld (23% vs. 44%); however, this was only statistically significant at the trend level (*p* = 0.08). Total score on the HCR-20 violence risk assessment tool was a significant predictor of outcome (OR = 0.80, 95% CI = 0.71, 0.90, *p* < 0.001), with patients who were discharged having a mean score of 21.3 compared to 26.8 for patients whose section was upheld. Those who were discharged additionally had significantly lower scores on both the Clinical scale (C scale) (OR = 0.61; 95% CI = 0.48, 0.79; *p* < 0.001) and Risk scale (R scale) (OR = 0.66; 95% CI = 0.52, 0.84; *p* = 0.001) of the HCR-20; no significant differences were observed on the HCR-20 Historical scale (H scale). Relative to patients who had displayed no aggression in an inpatient setting in the 12 months prior to census completion, those who were either verbally aggressive (OR = 0.21; 95% CI = 0.06, 0.76; *p* = 0.02) or physically violent (OR = 0.10; 95% CI = 0.02, 0.49; *p* = 0.005) were significantly less likely to be discharged by the tribunal panel. Similarly, individuals who had displayed agitated behaviour in the 12 months prior to the census were significantly less likely to be discharged (OR = 0.35; 95% CI = 0.13, 0.95; *p* = 0.04).

**Table 2:** Logistic Regression analyses examining behavioural and treatment-related predictors of outcome at MHRT

Predictor	Section Upheld (*n* = 51)	Discharge (*n* = 28)	OR	(95% CI)	*P* Value
Behavioural factors					
Index offence: *n* (%)					
Assault, ABH, GBH, wounding	27 (54.0)	14 (51.9)	(ref)	-----	-----
Murder, attempted murder, manslaughter	4 (8.0)	7 (25.9)	3.38	(0.84 to 13.52)	0.09
Sexual offence	12 (24.0)	2 (7.4)	0.32	(0.06 to 1.64)	0.17
Other	7 (14.0)	4 (14.8)	1.10	(0.28 to 4.42)	0.89
HCR-20 scores: mean (SD)					
Total score	26.8 (5.4)	21.3 (4.0)	0.80	(0.71 to 0.90)	<0.001
H Scale	14.5 (2.6)	14.2 (3.0)	0.95	(0.80 to 1.14)	0.60
C Scale	6.0 (2.7)	3.1 (1.9)	0.61	(0.48 to 0.79)	<0.001
R Scale	6.2 (2.5)	4.0 (2.0)	0.66	(0.52 to 0.84)	0.001
Substance use in an inpatient setting, past 12 months: *n* (%)	16 (32.0)	7 (25.9)	0.74	(0.26 to 2.12)	0.58
Aggression in an inpatient setting, past 12 months: *n* (%)					
No	17 (34.0)	20 (76.9)	(ref)	-----	-----
Verbal only	16 (32.0)	4 (15.4)	0.21	(0.06 to 0.76)	0.02
Attempted or actual physical violence	17 (34.0)	2 (7.7)	0.10	(0.02 to 0.49)	0.005
Inappropriate sexual behaviour in an inpatient setting, past 12 months: *n* (%)	10 (20.0)	2 (7.7)	3.00	(0.61 to 14.86)	0.18
Agitated behaviour in an inpatient setting, past 12 months: *n* (%)	37 (74.0)	13 (50.0)	0.35	(0.13 to 0.95)	0.04
Absconded or escaped, ever: *n* (%)	22 (44.0)	6 (23.1)	0.38	(0.13 to 1.11)	0.08
Treatment-related factors					
Engagement in psychological therapy: *n* (%)					
Engaged with good attendance	16 (33.3)	14 (53.8)	(ref)	-----	-----
Missed sessions or refused to engage	25 (52.1)	10 (38.5)	0.46	(0.16 to 1.28)	0.14
Not offered/unable to attend	7 (14.6)	2 (7.7)	0.33	(0.06 to 1.84)	0.20
Medication noncompliance, ever: *n* (%)	26 (52.0)	9 (36.0)	0.52	(0.19 to 1.39)	0.19
Depot: *n* (%)	13 (26.0)	6 (23.1)	0.85	(0.28 to 2.59)	0.78
Clozapine: *n* (%)	11 (22.0)	8 (30.8)	1.58	(0.54 to 4.59)	0.40

Note. OR: odds ratio; *n*: subgroup sample size; CI: confidence interval; SD: standard deviation; ABH: actual bodily harm; GBH: grievous bodily harm; HCR-20: Historical Clinical Risk-20; H Scale: historical scale; C Scale: clinical scale; R Scale: risk scale. Missing data: index offence (*n* = 2); HCR-20 total score (*n* = 7); HCR-20 H Scale (*n* = 7); HCR-20 C Scale (*n* = 7); HCR-20 R Scale (*n* = 7); substance use in an inpatient setting (*n* = 2); aggression in an inpatient setting (*n* = 3); inappropriate sexual behaviour in an inpatient setting (*n* = 3); agitated behaviour in an inpatient setting (*n* = 3); absconded or escaped, ever (*n* = 3); engagement in psychological therapy (*n* = 5); medication noncompliance, ever (*n* = 4); depot (*n* = 3); Clozapine (*n* = 3). For all variables, ‘past 12 months’, ‘current’, and ‘recent’ is relative to the time of the initial census

Treatment-related factors and their association with outcome at MHRT are presented in Table 2. Neither engagement in psychological therapy, medication compliance, depot antipsychotic treatment, nor clozapine treatment, were significant predictors of outcome at tribunal (*p* > 0.05).

### Multivariable analysis of dynamic predictors of outcome at MHRT

A multivariable logistic regression analysis was conducted to determine dynamic variables independently associated with outcome at MHRT after adjusting for the presence of other dynamic variables. Due to the small sample size (*n* = 79), the number of variables included was limited in order to maintain the validity of the model [37]. Thus, a more stringent entry criterion was employed where only variables which achieved a significance of *p* < 0.01 in univariable analyses were taken through to the multivariable analysis stage. Additionally, leave status was collapsed into a binary variable (no leave vs. any leave), in order to reduce the number of statistical parameters estimated by the model. The responsible clinicians’ recommendation was not advanced to the multivariable analysis stage as further univariable logistic regression analyses indicated that this factor was significantly associated with a number of the variables studied, including physical violence (OR = 0.08; 95% CI = 0.01, 0.66; *p* = 0.019), and the C (OR = 0.75; 95% CI = 0.60, 0.95; *p* = 0.015) and R (OR = 0.76; 95% CI = 0.60, 0.70; *p* = 0.026) scales of the HCR-20. This is likely due to the fact that the responsible clinician’s decision is based, at least in part, on factors entered as predictors in the multivariable model. As our interest was in factors that could be potentially targeted by the patient themselves (i.e., by changing their behaviour), we chose to exclude this variable from the multivariable model.

All variables entered into the multivariable model were first checked to see if the data met the assumption of collinearity, these tests indicated that multicollinearity was not a concern (Tolerance >0.10; VIF <10 for all variables entered into the final model). The final model (Table 3), which included the following dynamic variables: leave status, HCR-20 C scale, HCR-20 R scale, and recent aggression in an inpatient setting (dummy variables coding for both verbal aggression and physical violence were included in the multivariable model), correctly predicted outcome in 80.6% of cases (85.7% for section upheld and 72.0% for discharge) and was statistically significant (*χ* ^2^ = 32.98, *p* < 0.001). After entering all of the variables into the model simultaneously, scores on the HCR-20 C scale (OR = 0.68; 95% CI = 0.49, 0.96; *p* = 0.02) and attempted or actual physical violence (OR = 0.09; 95% CI = 0.01, 1.02; *p* = 0.05) were found to be significantly associated with outcome at tribunal after controlling for the effect of the other significant dynamic variables. Results were largely unchanged after adjusting for the lapse of time between baseline and tribunal in the multivariable model, although the effect of physical violence was reduced to trend level significance despite virtually no change in the odds ratio (OR = 0.10; 95% CI = 0.01, 22.50; *p* = 0.06).

**Table 3:** Multivariable logistic regression analysis examining dynamic predictors of outcome at MHRT

Predictor	OR	(95% CI)	*P* Value
HCR-20 Clinical scale	0.68	(0.49 to 0.95)	0.02
HCR-20 Risk scale	0.79	(0.57 to 1.09)	0.15
Aggression in an inpatient setting, past 12 months.			
No	(ref)	----	----
Verbal only	0.24	(0.04 to 1.29)	0.10
Attempted or actual physical violence	0.09	(0.01 to 1.02)	0.05
Any leave granted	1.48	(0.20 to 11.24)	0.70

Note. OR: odds ratio; CI: confidence interval; HCR-20: Historical Clinical Risk Management-20. Missing data: HCR-20 C Scale (*n* = 7); HCR-20 R Scale (*n* = 7); leave granted (*n* = 4); aggression in an inpatient setting (*n* = 3). For all variables, ‘past 12 months’ is relative to the time of the initial census

## Discussion

To our knowledge, this is the first prospective cohort study in the UK to examine dynamic factors associated with outcome at MHRT in a forensic psychiatric inpatient population. During the 2-year follow-up, 79 (59%) patients attended a tribunal, with 28 (35%) of these resulting in the patient being discharged. We found that unescorted community leave, a restricted MHA section and a recommendation of discharge from the patients’ responsible clinician were associated with a greater likelihood of discharge at tribunal. Whilst verbal aggression, physical violence, a recent episode of acute illness, a higher total score on the HCR-20 violence risk assessment, a higher score on the C and R scales of the HCR-20, and agitated behaviour were all negatively associated with discharge at tribunal. We also found that the HCR-20 C scale and attempted or actual physical violence uniquely predicted outcome after controlling for other dynamic variables.

Our findings are broadly consistent with those of previous retrospective studies conducted in both forensic and civil inpatient populations. In line with previous research [6, 13, 14, 17–22], demographic factors including sex, age, and ethnicity were not found to be significant predictors of outcome in the current study. Our finding that patients held on a restricted MHA section were significantly more likely to be discharged is consistent with the statistics for England and Wales [16], this is important as the majority of forensic patients in the UK are held on restricted MHA sections [26]. Our finding that a recent episode of acute illness was negatively associated with discharge was unsurprising and is consistent with Canadian research into dynamic variables associated with outcome at tribunal [7, 25]. Results with regards to the HCR-20 violence risk assessment are also consistent with previous forensic research; similar to Davoren and colleagues [21], we found that total scores on the HCR-20, along with scores on the C and R subscales, were significant predictors of outcome at tribunal. However, our findings indicate that the H scale of the HCR-20, assessing historical, criminogenic factors, was not associated with outcome.

A novel finding from our study was the association between patient leave status and outcome at tribunal, namely that patients with unescorted community leave at study commencement were significantly more likely to be discharged when compared to those patients with no leave. Whilst ours is the first study to have examined this specific factor, previous research has acknowledged that leave is an essential part of the rehabilitation of forensic patients and it is highly unlikely that a patient would be discharged without having been granted some form of leave [38].

Outcome at tribunal was not predicted by mental health diagnosis. Only one previous study has reported an association between mental health diagnosis and outcome at tribunal, with a diagnosis of mania [23] being associated with an increased chance of discharge at MHRT, other studies have reported no association between diagnosis and outcome at tribunal [17, 18, 20]. The ability of such factors to predict outcome at tribunal may depend on the demography of the sample and the clinical setting. In the current study, the majority of patients had a diagnosis of psychotic disorder, thus reducing variability in the sample, which may have precluded our ability to identify diagnosis as a significant predictor of outcome.

A number of studies have reported an association between psychopathic disorder [7, 13, 25] and outcome at MHRT, yet we found that the presence of psychopathy did not predict outcome at tribunal in the current study. Similarly, we observed no association between personality disorder and outcome at tribunal. This fits with our finding that the H Scale of the HCR-20, which specifically taps in to historical items including personality disorder, was not a significant predictor of outcome at tribunal. It is interesting that these factors, which are heavily weighted in violence risk assessment tools (due to the strong association between these factors and violence and recidivism [39]), do not appear to be particularly influential in the MHRT setting. This is surprising as the risk a patient poses to the public whilst in the community is an important consideration for the tribunal panel [40].

Previous research has found that when making a decision on outcome, tribunal panel members typically ask questions on a number of themes, including behaviour, cooperation with treatment, substance misuse, and activities on the ward [24]. Despite this, a number of dynamic factors related to these themes (i.e., substance use, inappropriate sexual behaviour, history of absconsion, and engagement in psychological therapy) were not found to significantly predict outcome in the current study. However, patients who exhibited verbal aggression, physical violence, or agitated behaviour in an inpatient setting in the 12 months prior to the census were significantly less likely to be discharged in comparison to those who had not displayed any aggressive or agitated behaviour. This is consistent with the findings of Hilton and Simmons [7], who demonstrated that patients who display institutional management problems (i.e., assault, rule-breaking, and lying) are significantly less likely to be discharged by the tribunal. Individuals who display violent or agitated behaviour in an inpatient setting are likely to be considered at higher risk of violence upon discharge to the community. As the risk a patient poses to the public is an important consideration for the tribunal [40], it would seem logical that those who are deemed a higher risk of violence will be less likely to be discharged.

Given the findings of previous studies in forensic populations [7, 25], it was surprising that outcome at MHRT was not predicted by medication compliance. Whilst the proportion of patients who had displayed medication noncompliance was higher among those whose section was upheld by the MHRT (52% vs. 36%), this was not statistically significant, although this may reflect a lack of statistical power due to the small sample in the current study which may have precluded our ability to identify significant associations. Consistent with previous research, we found that the strongest predictor of discharge at tribunal was the responsible clinicians’ recommendation. As one might expect, studies have found patients are significantly more likely to be discharged if their responsible clinician recommends discharge to the tribunal [7, 20, 25, 41]. This finding has been reported in civil and forensic populations, both within the UK and abroad.

Multivariable logistic regression was conducted, with all dynamic variables found to be significant at the *p* < 0.01 level in univariable analyses, in order to identify variables which remained significant predictors of outcome after controlling for the confounding effect of other significant dynamic factors. The model, which included leave status, HCR-20 C and R scale scores, and aggressive behaviour, was statistically significant, correctly predicting outcome in 80.6% of cases (85.7% for section upheld and 72.0% for discharge). Scores on the HCR-20 C scale and attempted or actual physical violence remained significantly associated with outcome at tribunal even after controlling for the effect of the other significant dynamic variables, indicating that these factors independently predicted outcome at MHRT. The responsible clinicians’ recommendation was not advanced to the multivariable analysis stage as exploratory analyses revealed that this factor was significantly related to a number of the variables studied, including physical violence and the C and R scales of the HCR-20. This is likely due to the fact that the responsible clinician’s decision is based, at least in part, on factors entered as predictors in the multivariable model and therefore this variable encapsulates the other variables studied. Further to this, the main aim of the current study was to examine the extent to which potentially targetable dynamic factors related to recent inpatient behaviour, cooperation with treatment, and substance misuse are associated with outcome at tribunal. As such, including this variable in the final multivariable model would likely reduce the clinical utility of the results (as our objective was to establish the factors underlying the responsible clinician’s decision with a view to identifying factors that the patient can potentially target in order to improve their chances of discharge). It is important to note, however, that even if these areas are successfully targeted by the patient, our findings imply that it is very unlikely that a patient would be successfully discharged if the responsible clinician does not also support their appeal.

Risk of violence is an important consideration, not only for MHRTs, but also for clinicians when making decisions on whether or not to grant a patient leave. Leave status was not a significant predictor of outcome in the multivariable model, which may be due to the effect of leave status being explained by scores on the HCR-20 C and R scales (i.e., indicating that leave status is merely an indication of violence risk which is captured more accurately by scores on the HCR-20). Indeed, a number of factors which are measured by the R scale of the HCR-20 may be used to guide decisions on leave status; for example, a patient may be less likely to be granted leave if they are noncompliant or have the potential to be exposed to destabilisers, both of which are captured by the HCR-20 R scale. The finding that scores on the HCR-20 C scale remained significantly associated with outcome at tribunal suggests that composite measures of risk (i.e., measures which take into account many different factors), are the strongest predictors of outcome at tribunal.

### Implications

This study has important implications for forensic psychiatric inpatients and their treating teams. Our findings imply that decisions at MHRT are not biased in terms of age, sex, ethnicity, mental health diagnosis, or even index offence. This provides reassurance for patients that the tribunal panel is not unduly influenced by historical patient-related factors that are outside of their control. Furthermore, our findings identify ways in which patients might improve their chances of being discharged at a MHRT; specifically, the factors which we have found to be significantly associated with outcome at MHRT could be presented to patients as areas for improvement. For example, items within the C and R scales of the HCR-20 violence risk assessment scale such as ‘Insight’ and ‘Treatment or supervision response’ are potentially targetable domains for patients. Indeed, a number of the SLaM forensic wards examined in the current study are already implementing a risk management intervention which focuses on the HCR-20 risk assessment, allowing patients to play an active role in rating their own risk assessments and understand how to manage their own risk. Groups such as these could play a fundamental role in enabling a patient to improve their prospects of discharge at tribunal by lowering their risk of violence [42].

Our finding that individuals who are discharged scored significantly lower on the HCR-20 violence risk assessment suggests that MHRT panels may be able to adequately interpret the information conveyed by standardised risk assessment tools. However, it is important that panel members are aware of the limitations with such violence risk assessments. A recent meta-analysis [43] found that whilst well-established violence risk assessment tools, including the HCR-20, were able to accurately predict those who were of low risk of violence, they were extremely poor at identifying those at high risk of violence (as indicated by a low positive predictive value and high negative predictive value). Fazel and colleagues concluded that the current evidence does not support the notion that violence risk assessment tools can be used as sole determinants on which panels make decisions regarding discharge. Thus, it is important that MHRT panels are aware of these limitations and do not rely too heavily on risk assessments such as the HCR-20.

### Limitations

Whilst the use of electronic medical records for research purposes has many advantages, such investigations are reliant on the accuracy and completeness of the data held within these records As such, some degree of misclassification (i.e., of the exposure and/or outcome variables) is possible. However, the majority of exposure variables examined in the current study were ascertained using a researcher-developed measure (the census form) which was completed by the patient’s clinical team; thus, variables not routinely-available within medical records were captured. In addition, the CRIS tool enabled us to search all available documents within these records to identify those relevant to our outcome measure (i.e., documents containing key words related to MHRT). The current study is additionally limited by the small sample which may have precluded our ability to identify significant associations between exposure variables and outcome at tribunal. Nonetheless, we were able to identify a number of factors that significantly predicted outcome (e.g., leave status, MHA section, and aggressive behaviour). The effect sizes that we have published in this study (i.e., odds ratios) will enable other research teams to conduct more robust sample size calculations prior to undertaking similar studies.

An important limitation of the present study is that the exposure variables were assessed at baseline (i.e., the time of the census) only and not proximally to outcome, therefore any differences (for example, possible improvements or declines) in the variables measured were not captured. The median length of time from baseline to the first tribunal was 222 days (range: 6–749). Thus, the association between dynamic variables (which by definition change over time) and outcome may have been attenuated.

Further to this, we were unable to examine factors outside of the ward environment which may have had a confounding effect on the decisions made by the MHRT, for example, a patient may be detained if there is no place available at local hostels for the patient to be discharged to. This study was conducted across low- and medium-secure forensic inpatient units within one London NHS trust; therefore the outcomes of the study are context dependent. However, a number of findings are consistent with previous research, suggesting that results are generalisable to an extent. A final limitation, that pertains to any observational study attempting to identify predictors of clinical decisions, relates to circularity. That is, patients may be more likely to appeal when they know the clinical team supports their discharge and likewise the team is more likely to support discharge when the variables that we have identified in this study are favourable: for this reason, we decided to exclude responsible clinician’s recommendation from the multivariable analysis. Nonetheless, this study identifies variables which, at the very least, clinical teams view as important factors that are prerequisite for successful discharge.

## Conclusions

The current study is the first in the UK to prospectively examine outcome at MHRT in a forensic inpatient population. By identifying factors associated with discharge at tribunal, the results of the primary analyses have important implications for forensic psychiatric patients and the clinical teams that provide care for them. Specifically, the results suggest that by reducing levels of agitated behaviour, verbal aggression and physical violence on the ward, working towards being granted unescorted community leave, and specifically targeting items on the HCR-20 risk assessment, patients may be able to improve their changes of discharge at a MHRT.

## Abbreviations

ABH: Actual bodily harm
BRC: Biomedical Research Centre
C scale: HCR-20 Clinical scale
CI: Confidence interval
CQC: Care Quality Commission
CRIS: Clinical Records Interactive Search
GBH: Grievous bodily harm
H scale: HCR-20 Historical scale
HCR-20: Historical Clinical Risk-20
HSCIC: Health and Social Care Information Centre
MHA: Mental health act
MHRT(s): Mental health review tribunal(s)
MHT: Mental health tribunal
MMPI: Minnesota multiphasic personality inventory
N: Total sample size
*n*: Subgroup sample size
NHS: National Health Service
OR: Odds ratio
PCL-R: Psychopathic checklist revised
PCL-SV: Psychopathic checklist screening version
PD: Personality disorder
R scale: HCR-20 Risk scale
RC: Responsible clinician
SCID-II: Structured clinical interview for DSM-IV Axis II personality disorders
SD: Standard deviation
SLaM: South London and Maudsley NHS Foundation Trust
SPSS: IBM statistical package for social scientists
UK: United Kingdom
USA: United States of America
